# Leber’s Hereditary Optic Neuropathy: The Mitochondrial Connection Revisited

**DOI:** 10.4103/0974-9233.75880

**Published:** 2011

**Authors:** Khaled K. Abu-Amero

**Affiliations:** Department of Ophthalmology, Ophthalmic Genetics Laboratory, College of Medicine, King Saud University, P. O. Box 245, Riyadh 11411, Saudi Arabia

**Keywords:** Leber’s Hereditary Optic Neuropathy, Mitochondrial DNA, Optic Neuropathy

## Abstract

Our current understanding of Leber’s hereditary optic neuropathy (LHON)-mitochondrial connection falls short of comprehensive. Twenty years of intensive investigation have yielded a wealth of information about mitochondria, the mitochondrial genome, the metabolism of the optic nerve and other structures, and the phenotypic variability of classic LHON. However, we still cannot completely explain how primary LHON mutations injure the optic nerve or why the optic nerve is particularly at risk. We cannot explain the incomplete penetrance or the male predominance of LHON, the typical onset in young adult life without warning, or the synchronicity of visual loss. Moreover, primary LHON mutations clearly are not present in every family with the LHON phenotype (including multigenerational maternal inheritance), and they are present in only a minority of individuals who have the LHON optic neuropathy phenotype without a family history. All lines of evidence point to abnormalities of the mitochondria as the direct or indirect cause of LHON. Therefore, the mitochondria-LHON connection needs to be revisited and examined closely. This review will attempt to do that and provide an update on various aspects of LHON.

## CLINICAL PRESENTATION OF LEBER’S HEREDITARY OPTIC NEUROPATHY

Leber’s hereditary optic neuropathy (LHON) was first described by the German ophthalmologist Theodore Leber (1840-1917),^1^ and is one of the most common inherited optic neuropathies, with a minimum disease prevalence of 1 in 30,000 in the North East of England.^2^ Similar figures have been reported in other Caucasian populations, with a LHON prevalence of 1 in 39,000 in the Netherlands and 1 in 50,000 in Finland.^3^,^4^

The age of onset of LHON is variable, ranging from 15 to 35 years, but individuals can become affected at any age between early childhood and over 60 years.^5^ The age of onset is slightly higher in females (19-55 years, average 31.3 years) than males (15-53 years, average 24.3).^5^ LHON is characterized by a marked gender bias, with over 80% of patients being male.^6^ However, neither gender nor mutational status significantly influences the age of onset of visual loss. The male to female ratio varies between mutations: 3:1 for m.3460G>A, 6:1 for m.11778G>A and 8:1 for m.14484T>C. The reason for the predominance remains undetermined. Approximately one in three cases have no clear family history.^2^

In the acute phase of the disease, patients typically present with painless visual blurring, with both eyes becoming affected either simultaneously (25% of cases) or sequentially (75% of cases) with a median inter-eye delay of 8 weeks.^7^ Rare cases of unilateral optic neuropathy in LHON have been reported, with the fellow remaining unaffected over a 16-year follow-up period.^8^ Visual acuity reaches a nadir 4 to 6 weeks after disease onset and is severely reduced to 6/60 or less. Ocular examination during the acute stage can reveal characteristic fundal changes that include a circumpapillary, telangiectatic micorangiopathy, swelling of the retinal nerve fiber layer, tortuosity of the retinal vasculature and disc hyperemia.^9^ However, it must be stressed that up to 20% of LHON cases will have a normal fundal appearance in the acute stage. The characteristic field defect in LHON is a steep-sided central or centrocecal scotoma and can be formally documented using Goldmann perimetry or a similar technique. In the chronic phase, the retinal nerve fiber layer gradually degenerates and the loss of retinal ganglion cell (RGC) axons results in a pale optic nerve (optic atrophy).^8^

In the majority of LHON patients, visual loss is permanent, but some patients experience visual recovery even several years following the onset of the disease.^10^ The chances of visual recovery are mutation-specific, being least with the m.11778G>A mutation that generally causes the most severe visual phenotype, and highest with the m.14484T>C mutation; the m.3460G>A mutation carries an intermediate prognosis. The improvement in visual parameters is not only restricted to visual acuity, but can also include the development of small islands of normal vision (fenestrations) within the central scotoma or^11^ a reversal of dyschromatopsia.^8^ However, significant visual recovery in LHON is rare and the majority of patients remain within the legal requirements for blind registration. There are also some additional phenotypes with additional clinical features including a multiple sclerosis-like syndrome, particularly in females with the m.11778G>A mutation (Harding’s disease).^12^ Additional phenotypes can also include cardiac conduction abnormalities,^13^ dystonia,^14^ palpitations, and syncope.^15^ Minor neurological features in LHON patients, including postural tremor, mild cerebellar ataxia, loss of deep tendon reflexes, and sensory neuropathy which have been labeled ‘LHON plus’^14^,^16^–^20^ have also been reported. LHON plus was reported in the presence^21^ and absence of mtDNA mutation.^22^

## WHY MITOCHONDRIA ARE IMPORTANT TO THE OPTIC NERVE?

**Figure 1:**
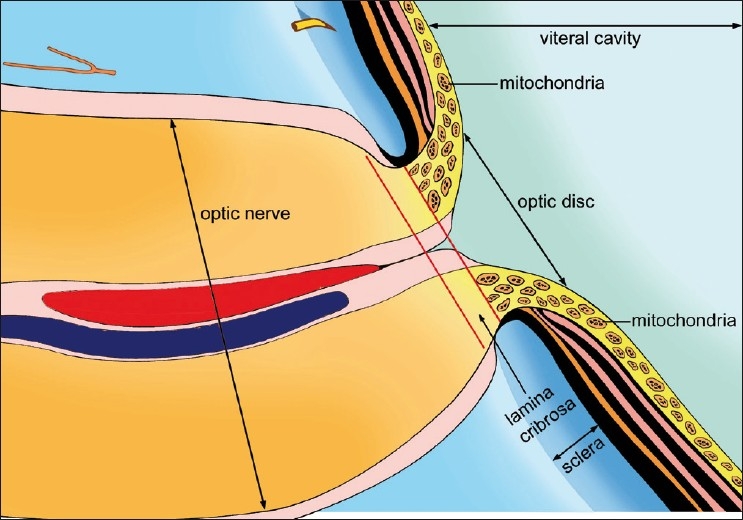
Digram showing the mitochondria location in the human eye

Mitochondria are important to the normal function of the optic nerve. There are high concentrations of mitochondria at the optic disk [Figure 1], which implies dependence on some aspect of mitochondrial function. A previous landmark study demonstrated that COX enzyme’s histochemical activity was undetectable in the myelinated optic nerve but was high in prelaminar and intralaminar regions, extending into the lamina cribrosa but halting abruptly in the retrolaminar position, which indicates that the presence of mitochondria may be limited to the unmyelinated portion of the optic nerve.^23^ Biochemical and cellular studies in LHON point to a partial defect of respiratory chain function that may generate either an ATP synthesis defect and/or a chronic increase of oxidative stress.^24^ Histopathological studies in LHON cases and a rat model mimicking Cuban epidemic of optic neuropathy (CEON) revealed a selective loss of retinal ganglion cells (RGCs) and the corresponding axons, particularly in the temporal-central part of the optic nerve.^25^ Anatomical peculiarities of optic nerve axons, such as the asymmetric pattern of myelination, may have functional implications on energy dependence and distribution of mitochondrial populations in the different sections of the nerve. Histological evidence suggests impaired axonal transport of mitochondria in LHON and in the CEON-like rat model, indicating a possible common pathophysiology for this category of optic neuropathies.^25^ Histological evidence of myelin pathology in LHON also suggests a role for oxidative stress, possibly affecting the oligodendrocytes of the optic nerves. The factors that render mitochondria in optic nerve cells unable to produce energy thereby producing cell death are largely unknown. One hypothesis is that cell death is actually programmed, and is the result of a random distribution of defective mitochondrial DNA during the normal mitotic division and aging process of cells.^26^

## MITOCHONDRIA AND APOPTOSIS

Apoptosis is the process of programmed cell death (PCD) that may occur in multicellular organisms. Biochemical events lead to characteristic cell changes (morphology) and death. These changes include blebbing, loss of cell membrane asymmetry and attachment, cell shrinkage, nuclear fragmentation, chromatin condensation, and chromosomal DNA fragmentation. Mitochondria play an important role in the regulation of apoptosis. They contain many pro-apoptotic proteins such as apoptosis-inducing factor, Smac/DIABLO, and cytochrome C. These factors are released from the mitochondria following the formation of a pore in the mitochondrial membrane called the permeability transition pore, or PT pore. These pores are thought to form through the action of the pro-apoptotic members of the bcl-2 family of proteins, which in turn are activated by apoptotic signals such as cell stress, free radical damage or growth factor deprivation. Mitochondria also play an important role in amplifying the apoptotic signaling from the death receptors, with receptor recruited caspase 8 activating the pro-apoptotic bcl-2 protein. Additionally, there is a growing body of evidence indicating that nitric oxide is able to induce apoptosis by helping to dissipate the membrane potential of mitochondria and therefore make it more permeable.^27^

## LEBER’S HEREDITARY OPTIC NEUROPATHY AND APOPTOSIS

There are several hints that pathology of the LHON syndrome may be connected with apoptosis. First, degeneration of the optic nerve, leading to the loss of vision, seems to appear in an apoptotic way (swelling of the mitochondria at the optic nerve head, release of cytochrome C).^28^ Second, studies of cybrids with the m.11778G>A and m.3460G>A mitochondrial DNA mutations show a higher sensitivity for Fas-induced apoptosis, in comparison to analogous cells without LHON mutation.^29^ That phenomenon can be explained by alterations in complex I. It is known that complex I and ubiquinone analogs play a regulatory role in the opening of the mitochondrial transition pore. Also metabolic stress produced by culture on galactose media (where cells have to rely only on oxygen respiration) lead to cell death and cybrids with the above mutations quickly switch to the apoptotic pathway.^30^ All these results suggest that sensitizing to apoptosis may be the actual role of LHON mutations in the disease development.

## MITOCHONDRIAL DNA MUTATIONS IN LEBER’S HEREDITARY OPTIC NEUROPATHY

In 1871 Leber^31^ described four families in which a number of young men experienced the abrupt loss of vision in both eyes either simultaneously or sequentially.^5^,^32^ Visual loss was profound, symmetric in the two eyes, and permanent.^33^ The inheritance pattern, which was initially thought to be X-linked and is now recognized to be maternal, became the single most important clue that LHON was due to a mtDNA abnormality.^34^ In 1988, Wallace and colleagues first reported the association of a G to A mutation at nucleotide position 11778 in the mitochondrial genome (ND4/G11778A) in nine pedigrees with a clinical diagnosis of LHON.^35^ The mutation converts a highly conserved arginine to histidine at codon 340 in the NADH dehydrogenase subunit 4 of complex I of the mitochondrial respiratory chain and is only rarely heteroplasmic.^36^,^37^ In relatively short order, a G to A point mutation at nucleotide position 3460 (ND1/G3460A)^38^,^39^ and a T to C mutation at nucleotide 14484 (ND6/T14484C)^40^,^41^ were reported in independent LHON pedigrees. These reports initiated the modern era in our understanding of LHON and other mitochondrial syndromes. These three mtDNA nucleotide changes are considered “primary” LHON mutations because each has been associated with the optic nerve injury of LHON even when occurring in isolation. The frequency of each has been studied extensively across the globe in individuals with multigenerational maternal inheritance of the LHON phenotype.^42^ They are thought to account for approximately 95% of LHON pedigrees of northern European descent.^42^ The incidence and distribution of primary LHON mutations have now been described in some detail in North America,^43^ Europe,^44^ Japan^45^ and Korea^46^ with the 11778 mutation accounting for 50-70% of individuals, the 14484 mutation for 10-15%, and the 3460 mutation for 8-25%.^47^ By contrast, a number of polymorphic mtDNA variants have been shown to influence the penetrance and clinical expression of these primary mutations.^48^–^50^ The pathologic role of the so called “secondary” LHON mutations remains unclear. What is known so far is that secondary LHON mutations cannot cause LHON in isolation, but the presence of these mutations in addition to one of the primary LHON mutation seems necessary for LHON development, at least in European lineages. A good example is the presence of the 11778 primary LHON mutation in conjunction with mitochondrial haplogroup J (defined mainly by the presence of three secondary LHON mutations, 4216, 13708 and 15257) suggesting a synergistic and deleterious interaction between primary LHON mutations and haplogroup J polymorphism(s).^51^,^52^

## MITOCHONDRIAL DNA MUTATIONS AND RESPIRATORY CHAIN DYSFUNCTION

Despite the evidence of increased reactive oxygen species (ROS) production and impaired respiratory chain functions the exact mode of retinal cell death remains unknown. Compared to control cybrids, LHON cybrid cell lines exhibit retarded cell growth when cultured on galactose medium,^53^ as well as show increased levels of apoptosis, particularly if they harbor either m.3460G>A or m.14484T>C. 2-deoxy-d-ribose-induced apoptotic response in lymphocytes also indicates a higher rate of apoptosis in LHON patients compared to controls.^54^ The increased cytochrome c release into the cytosol hallmarked by chromatin condensation and nuclear DNA laddering, by LHON cybrids indicates and involvement of mitochondria in the activation of the apoptotic cascade.^30^ After further investigation, it was found that cybrids growing in galactose medium underwent caspase-independent apoptosis due to a dramatic reduction of ATP. This apoptosis was mediated by apoptosis inducing factor (AIF or PDCD8) and endonuclease G (EndoG).^55^

## MOLECULAR GENETIC TESTING FOR LEBER’S HEREDITARY OPTIC NEUROPATHY

A strategy for genetic testing for LHON needs to be developed in the absence of primary LHON mutations in patients with clinically established LHON, or in the presence of secondary LHON mutations and the presence of other mitochondrial abnormalities such respiratory dysfunction,. If DNA testing for a clinical LHON is required, then the one starts by testing for the presence of one of the three primary LHON mutations. If positive, then the results should be reported. If the patient does not have any of the three LHON mutations and he/she is highly suspected of having LHON clinically, then one should test for the secondary LHON mutations. Care should be taken when interpreting the relevance of the so-called “secondary” mutations, which have been associated with LHON (e.g., m.4216T>C, m.13708G>A, m.15257G>A). These substitutions are also found in the general population albeit at a lower frequency^56^ and although they may modulate the risk of visual failure,^57^ they cannot cause LHON on their own. If neither primary nor secondary mutations are found, then direct sequencing of the mtDNA complex I genes, followed by complete mtDNA sequencing should be performed. This may reveal one of the previously identified, rarer, primary LHON mutations reported in MitoMap (http://www.mitomap.org/MITOMAP) or a novel (not previously described) mtDNA sequence variant. As for novel mtDNA sequence variants, pathogenic assessment should be carried out according to the criteria detailed previously.^58^ Another approach is to use simplified criteria taking into account the factors listed above to predict the pathologic status of mtDNA sequence changes that have not been previously described in patients with spontaneous optic neuropathies.^58^–^63^ These criteria are not meant to be comprehensive; rather, they are meant to eliminate the majority of mtDNA sequence changes that are merely polymorphisms. They include that the mtDNA sequence alteration should: (1) be non-synonymous (results in changing an amino acid); (2) not be reported in mitochondrial databases (e.g., MitoMap) or Medline-listed literature as a confirmed polymorphism; (3) not be present in ethnically matched healthy controls; (4) not be recognized as a haplogroup specific SNP; (5) occur in a genomic region of high interspecies conservation; and (6) be assessed by available databases (e.g., Polyphen; http://www.genetics.bwh.harvard.edu/pph/64 and SIFT, Sorting Intolerant From Tolerant, which predicts whether protein substitutions are tolerated; http://www.sift.jcvi.org/65) as potentially pathologic. Although detecting defects in mitochondrial respiratory function has its value in detecting mitochondrial abnormalities, biochemical assessment does not form part of the routine investigation of LHON patients. Heteroplasmy level is an important indicator in various mitochondrial disorders.^66^ However, in the settings of LHON, it had little or no value. This is because almost all mtDNA mutations associated with LHON are homoplasmic and measurements of heteroplasmy in the blood could lead to false positive results.^67^

## LEBER’S HEREDITARY OPTIC NEUROPATHY MANAGEMENT

### Treatment

There are currently no therapeutic interventions that have been shown to prevent the onset of visual symptoms or improve visual outcome in affected LHON carriers. A small, non-controlled trial suggested that oral administration of the quinone analog, idebedone, coupled with vitamin supplementation (B_12_ and C) may speed up visual recovery.^68^ However, more rigorous studies are required and through a multicenter collaboration, a phase II, double blind, randomized placebo-controlled trial is currently in progress to investigate the efficacy, safety, and tolerability of oral idebenone in LHON (Further details are available at http://www.lhon.ncl.ac.uk and http://www.lhon.de/). Recent studies have also explored the use of allotopically expressed mitochondrial superoxide dismutase to protect RGCs from oxidative stress by scavenging excess free radicals, providing the proof-of-principle that targeted gene therapy is a possible neuroprotective strategy in LHON.^69^

The long-term clinical management of acute LHON patients remains mainly supportive, with the provision of low-vision aids, occupational therapy and registration of affected individuals with the relevant social services. Based upon current epidemiological data on environmental risk factors in LHON and for general lifestyle reasons, LHON mutations carriers should be strongly advised to moderate their alcohol intake and abstain from smoking.

### Genetic counseling

Molecular diagnostic confirmation of LHON facilitates genetic counseling, but it is important for LHON carriers to be made aware that it is currently not possible to predict accurately whether or when they will become affected. Despite these limitations, the two main predictive factors for visual failure are age and gender. Males have a 50% lifetime risk of loss of vision compared to only 10% for females but these approximate figures can be further refined based upon the patient’s age. From published age-dependent penetrance data, we know that most patients experience visual loss in the first three decades of life and the probability of visual failure is low once past the age of 50

LHON mtDNA mutations are maternally inherited, which means that affected males cannot transmit the disorder to their offspring. Maternal relatives are, however, at risk of developing the disorder. There are well-established empirically derived recurrence risks for more distant relatives of an affected proband.^7^,^70^ As a rule of thumb, a woman who harbors a homoplasmic primary LHON mutation (i.e. 100% mutant) has a ~40% risk of having an affected son, and a ~10% risk of having an affected daughter. The presence of heteroplasmy (a mixture of mutant and wild-type mtDNA) is not uncommon in LHON families^71^ and theoretically reduces the recurrence risks. This makes genetic counseling complex for unaffected LHON carriers as there is a lack of robust prospectively acquired data in this situation.

### The future

Guy *et al*^72^ have proposed that mitochondrial genes affected by primary LHON mutations or under-expressed for unclear reasons in the setting of certain other spontaneous optic neuropathies can be augmented using a viral vector containing nuclear versions of the appropriate genes. In a series of preliminary experiments to investigate this concept, they made an immortal cell line from a patient with LHON and the 11778 mutation. They then created a version of the ND4 gene using the nuclear “alphabet”, attached the mitochondrial targeting sequence plus a FLAG tag, and used an adeno-associated viral vector to insert this gene into the cybrid cell nuclei. They showed that the coded protein was created and sent to mitochondria, that cell survival was increased three-fold, and that ATP production was normalized. All of this was accomplished without knowing exactly how the 11778 mutation affects ATP production or cell survival in tissue culture. This exciting new area of gene therapy research in humans will depend as much on our comprehensive understanding of mtDNA nucleotide changes as on the unique anatomy of the globe.

### Final remarks

Our current understanding of LHON-mitochondrial connection falls short of comprehensive. Twenty years of intensive investigation have yielded a wealth of information about mitochondria, the mitochondrial genome, the metabolism of the optic nerve and other structures, and the phenotypic variability of classic LHON. However, we still cannot completely explain how primary LHON mutations injure the optic nerve or why the optic nerve is particularly at risk. We cannot explain the incomplete penetrance or the male predominance of LHON, the typical onset in young adult life without warning, or the synchronicity of visual loss. Moreover, primary LHON mutations clearly are not present in every family with the LHON phenotype (including multigenerational maternal inheritance), and they are present in only a minority of individuals who have the LHON optic neuropathy phenotype without a family history. Finally, the phenotypic similarities between LHON and other spontaneous optic neuropathies may imply that they may also share mitochondrial risk factors.

The association between mitochondrial abnormalities and optic nerve damage is probably substantially larger than three primary LHON mutations and classic LHON. Now it seems likely that the LHON phenotype is somewhat broader that Leber himself described and that other mtDNA nucleotide changes (provisional LHON changes, and quite possibly others meeting the simplified criteria above) can be pathologic to the optic nerve. These nucleotide changes may be less damaging than primary LHON mutations, leading to less penetrance, less obvious maternal inheritance, and more interaction with other risk factors such as age, shape of the optic disk, atherosclerotic disease, and tendency to develop optic nerve inflammation. They may, nevertheless, play an important role in creating the metabolic landscape upon which other risk factors interact in certain individuals destined to experience a spontaneous optic neuropathy.
